# Captopril mitigates splenomegaly and myelofibrosis in the *Gata1* ^*low*^ murine model of myelofibrosis

**DOI:** 10.1111/jcmm.13710

**Published:** 2018-07-04

**Authors:** Seth J. Corey, Jyoti Jha, Elizabeth A. McCart, William B. Rittase, Jeffy George, Joseph J. Mattapallil, Hrishikesh Mehta, Mungunsukh Ognoon, Michelle A. Bylicky, Thomas A. Summers, Regina M. Day

**Affiliations:** ^1^ Division of Pediatric Hematology, Oncology & Stem Cell Transplantation The Massey Cancer Center at Virginia Commonwealth University Richmond VA USA; ^2^ Department of Pharmacology and Molecular Therapeutics Uniformed Services University of the Health Sciences Bethesda MD USA; ^3^ Department of Microbiology Uniformed Services University of the Health Sciences Bethesda MD USA; ^4^ Department of Anesthesiology Uniformed Services University of the Health Sciences Bethesda MD USA; ^5^ Neuroscience Graduate Program Uniformed Services University of the Health Sciences Bethesda MD USA; ^6^ Department of Pathology Uniformed Services University of the Health Sciences Bethesda MD USA

**Keywords:** drug repurposing, myelofibrosis, myeloproliferative neoplasms

## Abstract

Allogeneic stem cell transplantation is currently the only curative therapy for primary myelofibrosis (MF), while the JAK2 inhibitor, ruxolitinib. Has been approved only for palliation. Other therapies are desperately needed to reverse life‐threatening MF. However, the cell(s) and cytokine(s) that promote MF remain unclear. Several reports have demonstrated that captopril, an inhibitor of angiotensin‐converting enzyme that blocks the production of angiotensin II (Ang II), mitigates fibrosis in heart, lung, skin and kidney. Here, we show that captopril can mitigate the development of MF in the *Gata1* ^*low*^ mouse model of primary MF. *Gata1* ^*low*^ mice were treated with 79 mg/kg/d captopril in the drinking water from 10 to 12 months of age. At 13 months of age, bone marrows were examined for fibrosis, megakaryocytosis and collagen expression; spleens were examined for megakaryocytosis, splenomegaly and collagen expression. Treatment of *Gata1* ^*low*^ mice with captopril in the drinking water was associated with normalization of the bone marrow cellularity; reduced reticulin fibres, splenomegaly and megakaryocytosis; and decreased collagen expression. Our findings suggest that treating with the ACE inhibitors captopril has a significant benefit in overcoming pathological changes associated with MF.

## 1 INTRODUCTION

Primary myelofibrosis (MF) is a life‐threatening disease with a median survival of 3.5‐5.5 years.^1^ Allogeneic stem cell transplantation is currently the only curative therapy for primary MF,^2^ but, because of comorbidities and limited donor availability, its application is limited. Gene sequencing of patients with primary MF has revealed mutations in *JAK2*,* MPL* and *CALR* genes. To date, the JAK2 inhibitor ruxolitinib is approved only for palliation of symptoms associated with splenomegaly and fatigue,^3^ and there is no evidence that JAK2 inhibitors can reverse MF. Other JAK inhibitors have been evaluated in clinical trials but have displayed toxicities.^4^ Ruxolitinib therapy must frequently be withdrawn due to side effects, such as anaemia, thrombocytopenia and infections. Thus, novel, non‐toxic therapies are desperately needed for this molecularly heterogeneous disorder.

Primary MF is characterized by abnormal megakaryocytes, aberrant cytokine production and bone marrow failure with extramedullary haematopoiesis.^5^ Stem cell‐derived myeloproliferation and abnormal cytokine production lead to the dysregulation of megakaryocytes and fibrotic remodelling of the bone marrow.^6^ The degree of collagen fibrosis in the bone marrow can be correlated with the severity of primary MF.^6^

Several genetically engineered mouse models based on *JAK2*,* MPL* or *CALR* mutations are available to study MF.^7^, ^8^, ^9^ Patients with idiopathic MF were found to harbour reduced levels of the transcription factor GATA1 in megakaryocytes.^10^ GATA1 is a haematopoietic master transcription factor that provides regulation for both erythroid and myeloid lineages.^11^ Due to a deletion in the hypersensitive site of its promoter, which drives its transcription in megakaryocytes, GATA1 deficiency results in aberrant megakaryocytopoiesis resulting in hyperproliferative progenitors, defective terminal differentiation, impaired erythropoiesis and transient anaemia.^11^, ^12^ The *Gata1* ^*low*^ mouse strain has been especially useful to study MF because fibrotic remodelling of the bone marrow microenvironment also occurs.^13^, ^14^

A final common pathway that leads to MF is thought to involve aberrant regulation of TGF‐β1 and the subsequent deposition of reticulin and collagen.^15^ Recent work suggests that malignant and non‐malignant cells cooperate in this inflammatory process and subsequent fibrosis and that fibrocytes may play an important role in this process.^16^, ^17^ However, the identity of the cell types and the inflammatory cytokines directly responsible for myelofibrotic remodelling are not known, but might be important in developing more effective, non‐transplant therapies.

A number of studies have demonstrated the role of Ang II in fibrotic remodelling of the lung, heart, kidney, skin and liver.^18^, ^19^, ^20^, ^21^ It has been demonstrated in a number of animal models that inhibitors of angiotensin‐converting enzyme (ACE) can block or reverse fibrotic remodelling through the reduction in Ang II maturation.^22^, ^23^, ^24^, ^25^, ^26^ Therefore, we hypothesized that captopril, an ACE inhibitor, could reverse MF. We tested this hypothesis in the *Gata1* ^*low*^ mouse model of primary MF.

## 2 METHODS

### 2.1 Chemicals

Reagents were obtained from Sigma‐Aldrich (St. Louis, MO) except where indicated.

### 2.2 Animals and captopril treatment

All animal handling procedures were performed in compliance with guidelines from the National Research Council for the ethical handling of laboratory animals and were approved by the Uniformed Services University of the Health Sciences Institutional Animal Care and Use Committee. Male and female *Gata1* ^*low*^ and wild‐type CD1 mice were purchased from Jackson Laboratories (Bar Harbor, ME). Quantitative PCR confirmed low expression of *Gata1* (results not shown). The mice were crossed to a CD1 background as previously described to establish a line of homozygous mutant mice.^14^ Mice were kept in a barrier facility for animals accredited by the Association for Assessment and Accreditation of Laboratory Animal Care International. Mice were housed in groups of four. Animal rooms were maintained at 21 ± 2°C, 50% ± 10% humidity and 12‐hour light/dark cycle with commercial freely available rodent ration (Harlan Teklad Rodent Diet 8604, Frederick, MD, USA). Captopril (USP grade; Sigma‐Aldrich, St Louis, MO, USA) was dissolved in acidified water at 0.6 g/L and provided to animals starting at 10 months of age until 12 months of age, as previously described.^27^ An earlier study established the stability of captopril in acidified water.^28^ Based on previously measured volumes of water consumed per day by the mice, we determined that daily water consumption resulted in a dose of 79 mg/kg/d.^27^ Control animals received acidified water (vehicle) without captopril. Animals were killed at 13 months of age.

### 2.3 Blood cell analysis

Complete blood counts (CBC) with differentials were obtained using a Baker Advia 2120 Hematology Analyzer (Siemens, Tarrytown, NY, USA). Separate mice were used for each point (n = 5‐6 per group).

### 2.4 Histology and myelofibrosis scoring

Sternebrae, humeri and femurs were surgically removed from killed animals and fixed in 10% neutral formalin overnight. Tissues were paraffin blocked and stained using standard methods for haematoxylin and eosin (H&E), Masson's trichrome and Gomori reticulin stain by Histoserve (Germantown, MD). Stained slides were evaluated by a pathologist who was blinded to the identity of the treatment groups using a published system for scoring MF.^29^ Bone marrow sections were digitally scanned using the Zeiss Axio Scan and images for publication were produced with Zen Lite software (Carl Zeiss, USA).

### 2.5 Bone marrow and spleen cell isolation

Mice were killed with pentobarbital (10 mg/kg). Humeri and femurs were surgically removed from killed animals and flushed with sterile PBS. Spleens were smashed through 40 μmol/L cell strainer (Cell Treat, Pepperell, MA) using the plunger end of a small syringe. Cell strainer was rinsed with PBS (end volume of 30 mL) and cells were collected by centrifugation at 300× *g* for 10 minutes at room temperature. Red blood cells were lysed by resuspending bone marrow cells in 2 mL (1 minute incubation) or spleen cells in 5 mL of ACK lysis buffer (5 minutes incubation). Cells were then diluted in 20 mL PBS, washed twice and pelleted as before.

### 2.6 Cell staining and analysis

Cells isolated from spleen and bone marrow were resuspended in ~200 μL PBS and placed on 5 mL nylon cell strainer topped Falcon tubes (Corning Life Sciences, Corning, NY) and centrifuged for 10 minutes at 860× *g* at room temperature. Cells were resuspended in 100 μL PBS and transferred to Falcon 96 well clear V‐bottom not treated polypropylene storage microplates (Corning Life Sciences). Cells were then stained with LIVE/DEAD viability stain (Molecular Probes, Life Technology, Grand Island, NY) for 20 minutes in the dark, washed with staining buffer (0.5% FBS, 0.05% NaN_3_ in PBS) and pelleted by centrifugation for 5 minutes at 860× *g* at room temperature and subsequently blocked by 1 μL Fc Block (BD Bioscience, San Jose, CA) diluted in 99 μL staining buffer for 20 minutes on ice. Plates were centrifuged at 860× *g* for 5 minutes at room temperature, and supernatants were removed. After washing with 200 μL of staining buffer, the cells were stained with a cocktail containing: Brilliant Violet 605‐labelled CD45 (1:160, Cat#: 103140, BioLegend, San Diego, CA); allophycocyanin (APC)‐eFluor 780‐labelled CD115 (1:80, Ref#: 47‐1152‐82, Affymetrix eBioscience, San Diego, CA); and R‐Phycoerythrin (PE)‐labelled CD41 (1:160, Cat#558040, BD Bioscience, San Jose, CA) for 20 minutes on ice. After washing, cells were stained with anti‐biotin‐FITC (1:45, Miltenyi Biotec, San Diego) for 20 minutes on ice. The cells were washed, pelleted, resuspended in Perm/Wash buffer and analysed using a BD LSR II flow cytometer (BD Bioscience). Data analysis was carried out with FlowJo data analysis software version 10.1r5 (FlowJo, Ashland, Oregon). The gating strategy is shown in Figure S1.

### 2.7 Reverse transcription polymerase chain reaction (RT‐PCR)

Total RNA was extracted from cells isolated from bone marrow or spleen cells using phenol‐chloroform extraction with silicone lubricant using a modified protocol.^30^ Approximately 25 mg of tissue was homogenized in 1 mL of TRIzol reagent. After the addition of 200 μL of chloroform, 125 μL of RNase‐free water was added. Samples were added to prepared tubes and centrifuged at 8050 ***g*** for 15 minutes at 4°C. After recovery of RNA‐containing aqueous phase, one volume of 70% ethanol was added. RNA was obtained using the Qiagen RNeasy kit (Qiagen, Valencia, CA) for purification of total RNA from animal cells. RNA (500 ng) was used with the iScript cDNA kit (Bio‐Rad) for cDNA synthesis. Quantitative PCR was carried out on a CFX96 real‐time PCR detection system (Bio‐Rad), using 15 ng equivalent cDNA and SYBR Green qPCR master mix (Bio‐Rad). PCR reaction conditions were 3 minutes at 95.0°C, followed by cycles of 10 seconds at 95.0°C, 30 seconds at 55.0°C for 39 total cycles (Bio‐Rad CFX Manager 3.1 preloaded, CFX‐2stepAmp protocol). The following primers sequences were used for target amplification: collagen type III (Col III) (forward) 5′‐TCTGAAGCTGATGGGATCAA‐3′, (reverse) 5′‐TCCATTCCCCAGTGTGTTTAG‐3′; collagen type Ia2 (ColIa2) (forward) 5′‐GCAG‐ GTTCACCTACTCTGTCCT‐3′, (reverse) 5′‐CTTGCCCCATTCATTTGTCT‐3′; CD41 (forward) 5′‐AAGCTGAAGCCACAGTGGAG‐3′, (reverse) 5′‐TGGAGACCCATCTGTCCAA‐3′; CD61 (forward) 5′‐GCAAGTACTGTGAGTGCGATG‐3′, (reverse) 5′‐CGCAGTCCCCACAGTTACA‐3′; glyceraldehyde 3‐phosphate dehydrogenase (GAPDH) (forward) 5′‐CCGGGTTCCTATAAATAC ‐GGACTG‐3′, (reverse) 5′‐GTCTACGGGACGAGGCTGG‐3′. Relative gene expression to the housekeeping genes was calculated using the ΔΔCq method.^31^, ^32^

### 2.8 Statistical analysis

Statistical analysis was performed with GraphPad Prism 7 (San Diego, CA). Results are represented as means ± SEM. *P* values of < .05 were considered significant. Two‐way ANOVA with either Tukey's or Sidak's post hoc tests was used for multiple comparisons.

## 3 RESULTS

**Figure 1:**
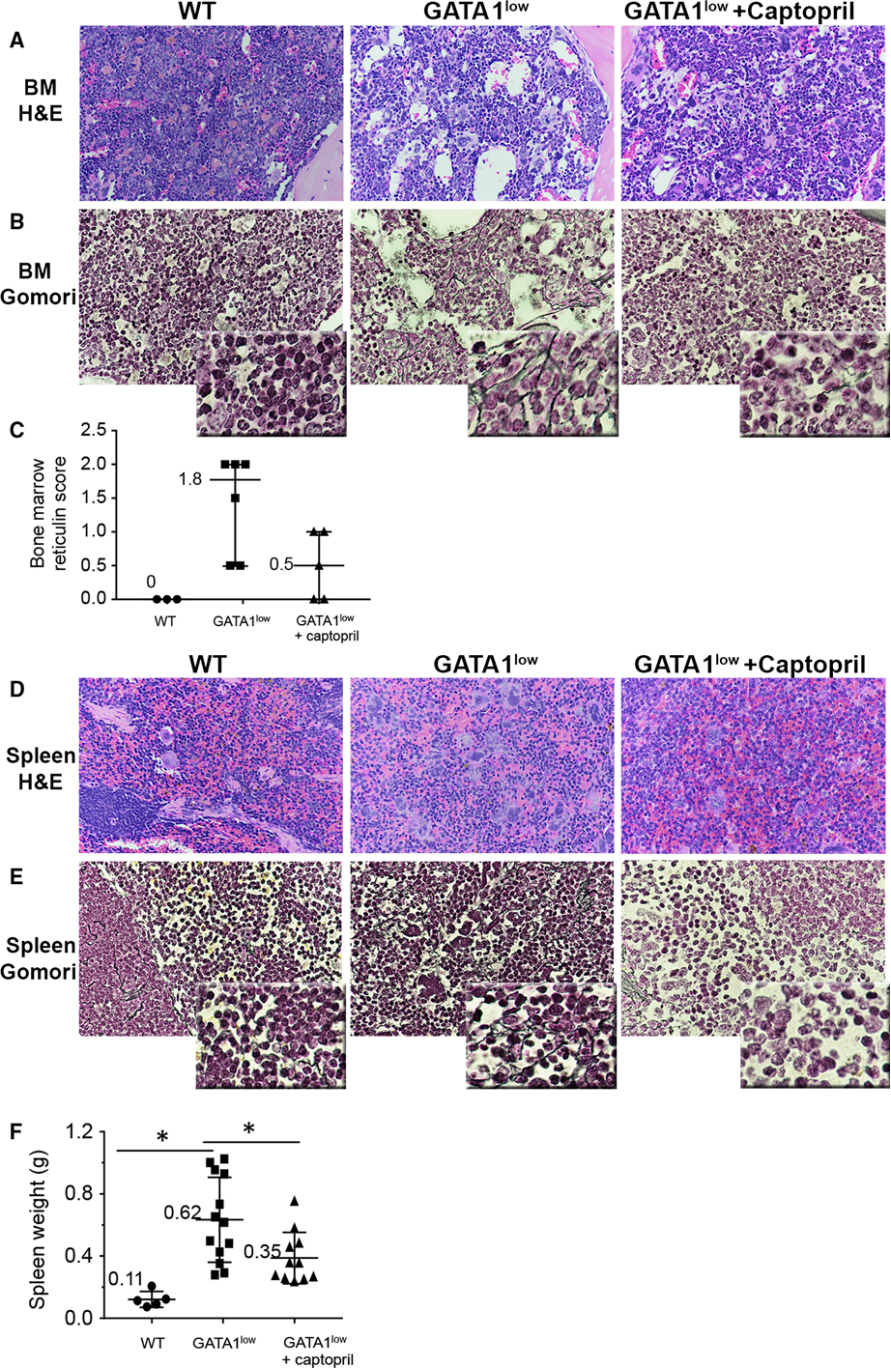
Effects of captopril administration on *Gata1* ^*low*^ mouse model of myelofibrosis. Wild‐type (wt) or *Gata1* ^*low*^ mice were treated from 10 to 12 mo with either captopril 72 mg/kg/d or vehicle in drinking water. Mice were killed at 13 mo, and tissues were harvested. (A) Haematoxylin and eosin staining of bone marrow from the humeri of the three mouse cohorts. Magnification is 40×. (B, C) Gomori staining of the same histological sections shows reticulin deposition in vehicle‐treated *Gata1* ^*low*^ mice. Under blinded conditions, a board‐certified pathologist (T.A.S.) scored bone marrow for reticulin. Captopril treatment resulted in decreased reticulin fibre score, with *P value < .05 (one‐tailed Mann‐Whitney test). (D) Haematoxylin and eosin staining of spleens from the three mouse cohorts. Magnification is 60×. (E) Gomori staining of the same histological sections shows reticulin deposition in vehicle‐treated *Gata1* ^*low*^ mice. (F) Spleens from the three mouse cohorts were weighed, with *P value < .05

To determine the efficacy of captopril in reversing MF, we evaluated morphologic and phenotypic changes in the *Gata1* ^*low*^ mouse model. Untreated *Gata1* ^*low*^ mice at 13 months of age exhibited classic features of marrow MF as compared to wild‐type CD1 mice (Figure 1A,B). Additional morphologic indications of fibrosis included cellular streaming and dilated sinuses. Megakaryocytes in the bone marrow of the *Gata1* ^*low*^ mice were abnormally present in patchy clusters and with paratrabecular distribution. The megakaryocytes in the *Gata1* ^*low*^ mice also displayed moderate megakaryocytic hyperplasia, with atypical morphology and enlarged bulbous nuclei compared with wild type. The reticulin score averaged 1.8 out of 3 in the *Gata1* ^*low*^ mice, in contrast to wt mice that scored reticulin as 0 (normal) (Figure 1C, *P* value < .05 by one‐tailed Mann‐Whitney test). Captopril treatment for 2 months, from 10 to 12 months of age, reduced the severity of bone marrow fibrosis at 13 months of age, with only focal and patchy cellular streaming and rare dilated sinuses (Figure 1A‐C). Captopril‐treated mice had only mild megakaryocytic hyperplasia, with scattered morphologically abnormalities and displayed only focal megakaryocytic clusters compared with untreated *Gata1* ^*low*^ mice. Treatment with captopril reduced the averaged reticulin score to 0.5 in the *Gata1* ^*low*^ mice.

**Figure 2:**
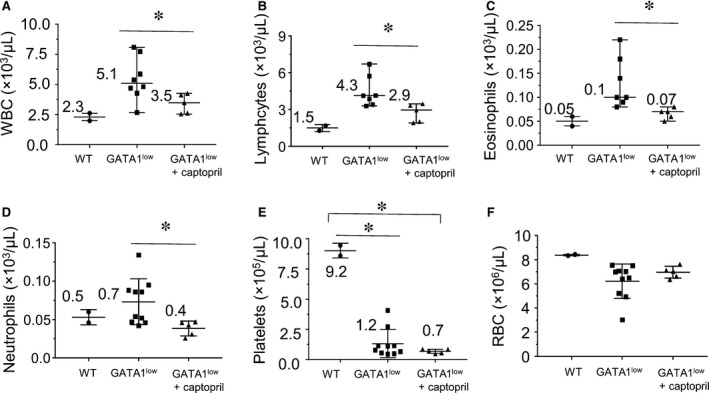
Effects of captopril administration on peripheral blood counts. Wild‐type (wt) or *Gata1* ^*low*^ mice were treated from 10 to 12 mo with either captopril 72 mg/kg/d or vehicle in drinking water. Mice were killed at 13.5 mo, and tissues were harvested. Complete blood cell counts with differentials were obtained. (A) white blood cells (WBC); (B) lymphocytes; (C) eosinophils; (D) neutrophils; (E) platelets; and (F) red blood cells (RBC). Means are indicated; *indicates *P* < .05

Levels of megakaryocytes and extramedullary haematopoiesis were compared in the spleens of wt and untreated and captopril‐treated *Gata1* ^*low*^ mice. Histologically, the untreated *Gata1* ^*low*^ mice demonstrated significant extramedullary haematopoiesis with increased numbers of enlarged atypical megakaryocytes which were present, in some areas, in large aggregates and sheets. The captopril‐treated *Gata1* ^*low*^ mice demonstrated moderate amounts of extramedullary haematopoiesis with reduced numbers of atypical megakaryocytes (Figure 1D,E). Consistent with previous reports of splenomegaly in *Gata1* ^*low*^ mice, we observed that the splenic weight was increased sixfold in untreated *Gata1* ^*low*^ mice as compared to wt CD1 mice (*P* value < .05). Captopril treatment for 2 months induced a ~2‐fold decrease (*P* < .05) in splenic weight in *Gata1* ^*low*^ mice as compared to untreated *Gata1* ^*low*^ mice (Figure 1F). Peripheral blood counts were studied in captopril‐treated and untreated *Gata1* ^*low*^ mice and their wild‐type littermates. As shown in Figure 2, captopril treatment normalized white blood cells (WBC), lymphocytes, eosinophils and neutrophils compared with untreated *Gata1* ^*low*^ mice (Figure 2A‐D). Interestingly, captopril treatment did not ameliorate the platelet count (Figure 2E) or mean platelet volume (data not shown). *Gata1* ^*low*^ mice have been demonstrated to have reduced platelet numbers, believed to be due to MK dysfunction; although captopril reduced the numbers of MKs, the remaining MKs were still not functional for platelet production. We did not observe significant reduction in red blood cells (RBC) in the *Gata1* ^*low*^ mice at this time‐point (Figure 2F); this is consistent with previous findings indicating that the onset of anaemia is usually later than 13 months (REF). These data suggest that captopril's effects serve to normalize the levels of a number of blood cells.

**Figure 3:**
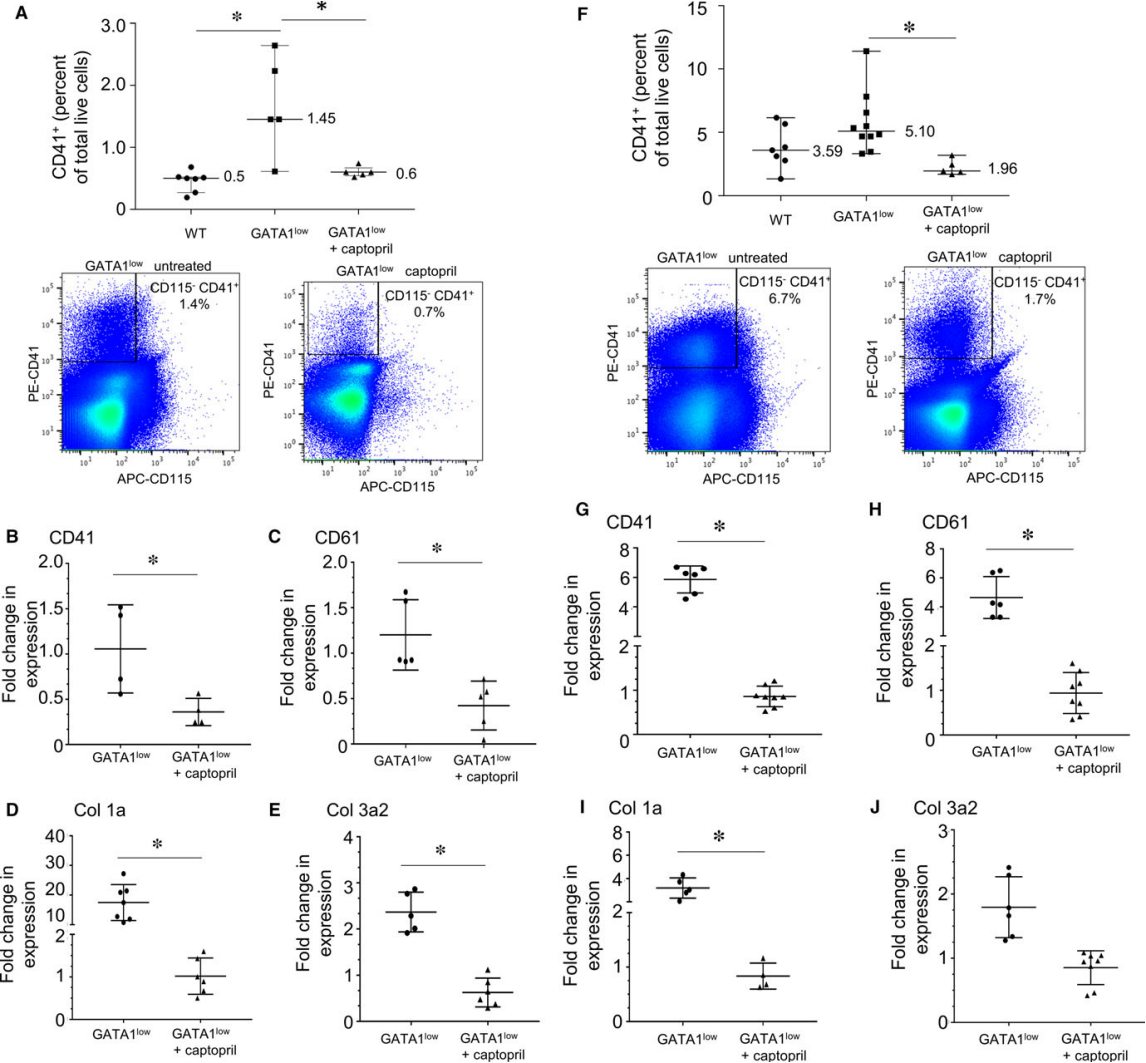
Effects of captopril administration of megakaryocytes and collagen. Wild‐type (wt) or *Gata1* ^*low*^ mice were treated from 10 to 12 mo with either captopril 72 mg/kg/d or vehicle in drinking water. Mice were killed at 13.5 mo, and tissues were harvested. (A) Flow cytometric analysis was performed on femur bone marrow cells to measure percentage of CD45^+^ cells expressing CD41^+^ cells. Representative FACS data are presented for wt, *Gata1* ^*low*^‐untreated mice and *Gata1* ^*low*^ captopril‐treated mice. (B‐E) qPCR of mRNA isolated from bone marrow of *Gata1* ^*low*^ mice treated ± captopril, as described above. Interrogated transcripts were *CD41*,* CD61*,* Col 1a* and *Col 3a*. Data show *Gata1* ^*low*^ qPCR transcript levels from untreated mice compared to the ratio of *Gata1* ^*low*^ transcript levels from mice treated with captopril relative to *Gata1* ^*low*^‐untreated mice. *P value .05. (F) Flow cytometric analysis was performed on spleen‐derived cells to measure percentage of CD45^+^ cells expressing CD41^+^ cells. Representative FACS data are presented for wt, *Gata1* ^*low*^‐untreated mice and *Gata1* ^*low*^ captopril‐treated mice. (G‐J) qPCR of mRNA isolated from spleens of *Gata1* ^*low*^ mice treated ± captopril, as described above. Interrogated transcripts were *CD41*,* CD61*,* Col1a* and *Col3a*. Data show *Gata1* ^*low*^ qPCR transcript levels from untreated mice compared to the ratio of *Gata1* ^*low*^ transcript levels from mice treated with captopril relative to *Gata1* ^*low*^‐untreated mice. *P value .05

We investigated the possible mechanism of action of captopril in the bone marrow and spleen. Flow cytometric analysis of murine mononuclear cells demonstrated a ~3‐fold increase in the frequency of CD115^−^/CD41^+^ megakaryocytes of total live cells in the bone marrow of *Gata1* ^*low*^ mice compared to wt CD1 mice, from 0.5% to 1.45% (*P* < .05) (Figure 3A). Captopril treatment reduced the number of megakaryocytes to 0.6% of total live cells (*P* < .05). These results were confirmed by qRT‐PCR detection of CD41 and CD61 markers, which were decreased ~3‐fold and 2‐fold, respectively, in *Gata1* ^*low*^ mice treated with captopril as compared to untreated mice (*P* < .05) (Figure 3B,C). There was reduced expression of both *Col1a* and *Col3a2*, which decreased ~15‐fold and ~4‐fold, respectively (*P* < .05) (Figure 3D,E).

Because of the observed changes in spleen histology and weight from captopril administration, we investigated the effect of captopril on megakaryocytes and collagen in the spleens of *Gata1* ^*low*^ mice. Flow cytometric analysis also showed that *Gata1* ^*low*^ mice had a trend towards higher levels of splenic megakaryocytes as compared to wt CD1 mice (Figure 3F), although this did not reach significance. We observed a ~2‐fold decrease in the frequency of megakaryocytes as a percentage of total live cells in response to captopril treatment (*P* < .05). This decrease in megakaryocytes as determined by FACS was also reflected in qRT‐PCR detection of CD41 and CD61 markers, which decreased ~6‐fold and ~5‐fold, respectively, in captopril‐treated *Gata1* ^*low*^ mice (*P* < .05) (Figure 3G,H). Histological observations of the spleen suggested that captopril induced a decrease in collagen fibres, so we investigated collagen expression levels in the spleen. qPCR analysis showed a ~4‐fold reduction in the level of *Col1a* expression (*P* < .05) and a trend towards reduced *Col3a2* expression, although this did not reach significance (Figure 3I,J).

## 4 DISCUSSION

MF is a rare myeloproliferative neoplasm characterized by hyperproliferation of abnormal megakaryocytes, deposition of collagen and reticulin in the bone marrow and splenomegaly associated with extramedullary haematopoiesis. Here we demonstrate that in the *Gata1* ^*low*^ murine model of spontaneous myelofibrosis a 2‐month administration of captopril, an ACE inhibitor commonly used for the treatment of systemic hypertension, decreased bone marrow megakaryocytic hyperplasia and marrow fibrosis. Furthermore, we show that captopril administration reduced the deposition of reticulin and collagen in the bone marrow of *Gata1* ^*low*^ mice histochemically, correlating with reduced collagen 1a and 3a synthesis at the mRNA level in marrow. These findings were also reflected in data demonstrating that captopril treatment decreased extramedullary haematopoiesis in the spleen, as indicated by both decreased splenic mass and morphologic changes in the spleen and attenuated collagen 1a and 3a mRNA in the spleen. As megakaryocytosis is believed to contribute to MF, the resolution of megakarycytosis is a critical event for reversal of the disease.

The fibrotic alterations observed in myelofibrotic bone marrow are similar to fibrotic alterations identified in other organs with regard to the up‐regulation of abnormal extracellular matrix proteins, most notably collagens, and the loss of normal cell types of the tissue.^33^, ^34^, ^35^ Thus, we hypothesized that inhibition of fibrotic signalling pathways would result in a reduction in bone marrow abnormalities of MF, possibly mitigating the disease. ACE and Ang II are believed to play a causative role in fibrosis of a number of tissues,^36^, ^37^, ^38^, ^39^, ^40^, ^41^, ^42^ and captopril and other ACE inhibitors or angiotensin receptor blockers (ARBs) were demonstrated to reduce fibrotic remodelling in a number of rodent models of fibrosis in tissues including kidney, lung, skin, liver and heart.^22^, ^43^, ^44^, ^45^, ^46^, ^47^, ^48^, ^49^, ^50^ In many of these studies, prevention of fibrosis by ACE inhibitors or ARBs was accompanied by reduced levels of myofibroblasts, attenuated collagen production, decreased inflammation and the preservation of normal tissue function and structure. Findings in animal model systems promoted the study of ACE and ARBs for treatment of human fibrotic disease. Clinical trials have also demonstrated that ACE inhibitors reduce medical radiotherapy‐induced kidney and lung fibrosis.^51^, ^52^, ^53^, ^54^, ^55^ The mechanism(s) by which ACE inhibitors and Ang II receptor antagonists inhibit fibrotic remodelling are not completely understood. Our observations in the *Gata1* ^*low*^ murine model of MF are consistent with previous studies showing a mitigation of fibrotic remodelling by ACE inhibitors, including a reduction in abnormal collagen deposition and the restoration of more normal tissue architecture.

Our study also demonstrated a marked reduction in abnormal megakaryoctyes in the *Gata1* ^*low*^ mice after captopril treatment. Ang II, as a part of the renin‐angiotensin system, is a master regulator of blood pressure and blood volume homeostasis.^56^ This system has also been demonstrated to regulate cell proliferation and differentiation of specific haematopoietic lineages.^57^ Ang II was shown to be required for normal myelopoiesis and erythropoiesis.^58^ ACE knockout mice, in which Ang II levels are 10‐fold lower than in wt mice, have several myelopoietic abnormalities resulting in a reduction in normal, mature macrophages and have an accumulation of myeloblasts and myelocytes.^59^ Additionally, Ang II peptide administration in mice was shown to increase levels of megakaryocyte precursors and megakaryocytes in the blood after radiation exposure.^60^ Findings from our laboratory and others indicated that captopril increased survival from radiation‐induced haematopoietic injuries suggesting that ACE inhibition can also reduce injuries to the haematopoietic system.^27^, ^61^, ^62^, ^63^ ACE inhibitors were also shown to cause a reduction in granulocyte colony‐forming and erythroid burst‐forming units which were accompanied by an increase in undifferentiated cells, including granulocyte, erythroid, macrophage and megakaryocyte colony‐forming units (CFU).^58^, ^64^ Investigation of the direct effects of Ang II on bone marrow colony formation demonstrated that the addition of Ang II to bone marrow cultures resulted in the stimulation of immature CFU granulocyte/macrophage and CFU granulocyte/erythrocyte/monocyte/megakaryocyte under pan‐myeloid culture conditions.^65^ However, it was later demonstrated that the addition of Ang II did not affect CFU megakaryocyte colony formation in a lineage assay in culture.^59^

Captopril's ability to reverse fibrosis in this murine model is novel and future studies are needed to assess its feasibility for clinical use. The JAK2 inhibitor ruxolitinib reduces splenic haematopoiesis but does not reverse MF in the *Gata1* ^*low*^ mice,^66^ and ruxolitinib is currently approved by the Food and Drug Administration (FDA) only for palliation of splenomegaly and MF‐associated symptoms. Results of several clinical trials have thus far failed to demonstrate its reversal of fibrosis.^67^ Because captopril is a FDA‐registered drug with widespread use, low cost and little toxicity, our studies provide compelling evidence to initiate a phase I/II trial in patients with primary MF aimed at reducing marrow fibrosis, replacement blood product usage and MF‐associated symptoms. The human equivalent dose to 110 mg/kg/d captopril (0.55 g/L in the drinking water) is ~330 mg/d.^68^ Captopril's maximally tolerated dose of 500 mg/d ,^69^ which makes our dosage feasible. Our initial treatments with captopril were based on our findings of prevention of bone marrow injury by total body irradiation in mice.^27^, ^63^ We have since found that reduction in captopril levels to as low as 13 mg/kg/d is sufficient for the prevention of radiation‐induced bone marrow injury in mice (R.M. Day, unpublished findings). We wish to repeat our work in the *Gata1* ^*low*^ myelofibrosis model also using this reduced dosage of captopril. In addition, we are currently investigating the molecular mechanism of captopril‐mediated reduction in fibrosis and identifying the cytokine(s) responsible.

## CONFLICT OF INTEREST

All authors confirm that there is no conflict of interest.

## Supporting information


**Figure S1** Gating strategy for megakaryocytes from isolated PBMC. Total lymphocytes were gated based on forward versus side scatter followed by gating live cells that were negative for the LIVE‐DEAD marker. CD41+CD115‐ cells were gated based on the differential expression of CD41 and CD115.
