# Supplementary material for: Captopril mitigates splenomegaly and myelofibrosis in the Gata1 low murine model of myelofibrosis
Source: J Cell Mol Med. 2018 Jul 4;22(9):4274–82. doi: 10.1111/jcmm.13710 (PMC6111823; doi:10.1111/jcmm.13710)
Supplement: Supplementary file 1 — Figure S1 Gating strategy for megakaryocytes from isolated PBMC. Total lymphocytes were gated based on forward versus side scatter followed by gating live cells that were negative for the LIVE‐DEAD marker. CD41+CD115‐ cells were gated based on the differential expression of CD41 and CD115. [file JCMM-22-4274-s001.docx]

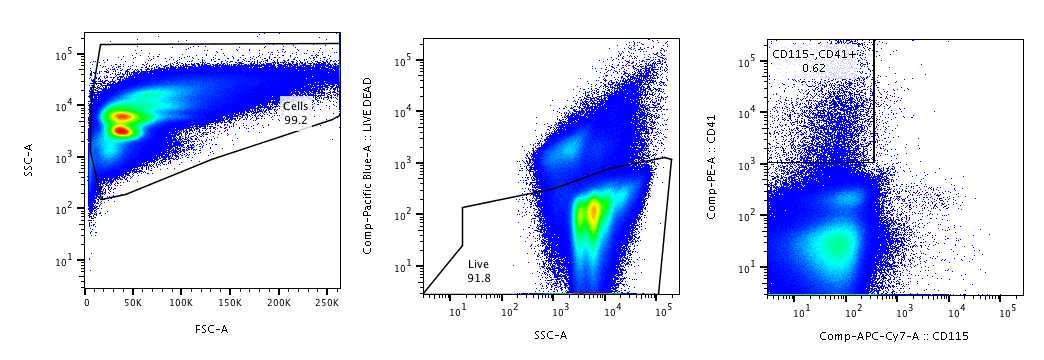


Supplemental figure 1: Gating strategy for megakaryocytes from isolated PBMC. Total lymphocytes were gated based on forward versus side scatter followed by gating live cells that were negative for the LIVE-DEAD marker. CD41+CD115- cells were gated based on the differential expression of CD41 and CD115.
